# Histological image classification using biologically interpretable shape-based features

**DOI:** 10.1186/1471-2342-13-9

**Published:** 2013-03-13

**Authors:** Sonal Kothari, John H Phan, Andrew N Young, May D Wang

**Affiliations:** 1 Department of Electrical and Computer Engineering, Georgia Institute of Technology, Atlanta, GA, USA; 2 Department of Biomedical Engineering, Georgia Institute of Technology and Emory University, Atlanta, GA, USA; 3 Pathology and Laboratory Medicine, Emory University, Atlanta, GA, USA; 4 Grady Health System, Atlanta, GA, USA

## Abstract

**Background:**

Automatic cancer diagnostic systems based on histological image classification are important for improving therapeutic decisions. Previous studies propose textural and morphological features for such systems. These features capture patterns in histological images that are useful for both cancer grading and subtyping. However, because many of these features lack a clear biological interpretation, pathologists may be reluctant to adopt these features for clinical diagnosis.

**Methods:**

We examine the utility of biologically interpretable shape-based features for classification of histological renal tumor images. Using Fourier shape descriptors, we extract shape-based features that capture the distribution of stain-enhanced cellular and tissue structures in each image and evaluate these features using a multi-class prediction model. We compare the predictive performance of the shape-based diagnostic model to that of traditional models, i.e., using textural, morphological and topological features.

**Results:**

The shape-based model, with an average accuracy of 77%, outperforms or complements traditional models. We identify the most informative shapes for each renal tumor subtype from the top-selected features. Results suggest that these shapes are not only accurate diagnostic features, but also correlate with known biological characteristics of renal tumors.

**Conclusions:**

Shape-based analysis of histological renal tumor images accurately classifies disease subtypes and reveals biologically insightful discriminatory features. This method for shape-based analysis can be extended to other histological datasets to aid pathologists in diagnostic and therapeutic decisions.

## Background

We develop an automatic histological image classification system that uses biologically interpretable shape-based features. These features capture the distribution of shape patterns, described by Fourier shape descriptors, in different stains of a histological image. We use this system to classify hematoxylin and eosin (H&E) stained renal tumor images and assess its classification performance by comparing it to methods based on textural, morphological, and topological features.

**Figure 1:**
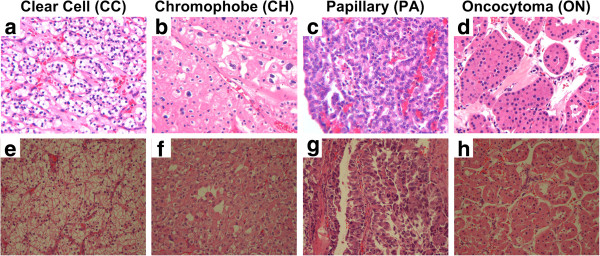
**Example images of four H&E stained histological renal tumor subtypes in datasets A (a-d) and B (e-h).** Among four subtypes, three are renal cell carcinoma (RCC) subtypes: (**a** and **e**) clear cell, (**b** and **f**) chromophobe, and (**c** and **g**) papillary. The fourth subtype is a benign renal (**d** and **h**) oncocytoma tumor.

The application of this system to cancer is important because, despite progress in treatment (e.g., early diagnosis, reduction of mortality rates, and improvement of survival), cancer is still a major health problem in the United States. Specifically, it is estimated that there were 60,920 new kidney and renal pelvis cancer cases in the United States in 2011, resulting in 13,120 deaths [1]. Successful prognosis or treatment of renal cell carcinoma (RCC) depends on disease subtype, each of which exhibits distinct clinical behavior and underlying genetic mutations [2]. Thus, it is important to accurately determine the subtype of an RCC patient from among the most common subtypes: clear cell (CC, 70% of cases), papillary (PA, 15%), and chromophobe (CH, 5%) [3]. In addition, it is also important to identify benign renal tumors, the most common of which are the renal oncocytomas (ON, 5% of cases). Figure 1 shows typical examples of H&E-stained renal tumor images. Pathologists, guided by the World Health Organization (WHO) system, manually classify renal tumors using light microscopy based on typical features [3]. Even though the WHO system is capable of classifying typical examples, some cases are more difficult. For example, ON and CH are often confused because both have granular cytoplasm. CH and CC can also be confused because both have prominent cell membranes. Moreover, there are two reported subtypes of PA that have varying visual appearance [3]. Thus, a pathologist’s diagnosis may be subjective.

Over the last decade, several automatic or automated systems have been developed to aid histological cancer diagnosis and to reduce subjectivity. All of these systems attempt to mimic pathologists by extracting features from histological images. Some important features include color, nuclear shape, fractal, textural gray-level co-occurrence matrices (GLCM), wavelets, and topological, among others [4,5]. Several diagnostic systems for renal cell carcinoma (RCC) are good examples of the utility of these features. For example, Chaudry et al. proposed a system using textural and morphological features with automated region-of-interest selection for RCC subtype classification [6,7]. Waheed et al. performed a similar analysis but included fractal as well as textural and morphological features [8]. Choi et al. extended the morphological analysis to three-dimensional nuclei and applied their system to RCC grading [9]. In addition to morphological features, Francois et al. used cell kinetic features in their RCC grading system [10]. Finally, Raza et al. used a scale invariant feature transform (SIFT) method to classify RCC subtypes [11]. Despite the success of these systems in terms of diagnostic accuracy, widespread use of these systems is limited by a lack of feature interpretability. Some researchers have provided visual interpretation of features. For example, some topological features have been related to the amount of differentiation in varying cancer grades [12]. In contrast, pathologists may not be receptive to, or confident in, features such as wavelet or fractal representations of images because they are not easy to interpret biologically. Moreover, most existing systems exploit morphological properties of nuclear shapes and ignore cytoplasmic and glandular structures despite evidence of their utility [13]. Thus, methods based on a holistic view of shapes and colors may more accurately reflect the process by which a pathologist interprets a renal tumor image [3].

Fourier shape descriptors, described by Kuhl and Giardina [14] have been reported to be very useful as shape descriptors. They are highly robust to high frequency noise because of their ability to reject higher harmonic shape descriptors. Researchers have used Fourier shape descriptors for various medical imaging applications, including shape-based vertebral image retrieval [15], and classification of breast tumors [16]. The medical images involved in these studies typically have definite shapes with consistent landmarks. In addition, researchers have used Fourier shape descriptors for analyzing the shapes of nuclear structures [17-19]. Histological images, however, lack such landmarks and they tend to exhibit multiple highly variable shapes. As such, it is difficult to compare histological images using common techniques such as template matching with an image atlas [20] or using shape-based similarity measures after registration of the shapes in a histological image [21]. Therefore, in order to characterize and compare histological images in terms of shapes, we quantify the distribution of shape patterns in an image using Fourier shape descriptors.

**Figure 2:**
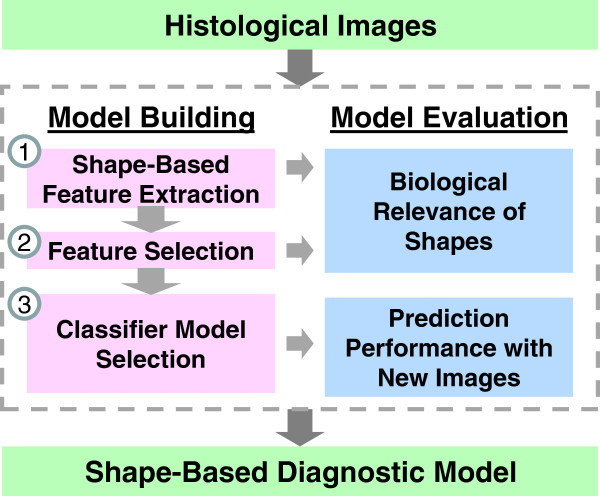
**Building and evaluating a shape-based diagnostic model using histological images.** We use three steps to derive a shape-based diagnostic model from histological images: 1) shape-based feature extraction (including automatic color segmentation, individual shape descriptor extraction, and discretization), 2) feature selection using the minimum redundancy-maximum relevance (mRMR) method, and 3) classifier model selection using cross-validation to identify optimal model parameters (i.e., feature size, Fourier shape descriptor harmonics, and SVM parameters). We evaluate the selected features and the classifier model by examining the biological relevance of the top selected features and by classifying independent images (using nested cross-validation).

We use three steps to build a diagnostic model from a set of histological images: (1) shape-based feature extraction, (2) feature selection, and (3) classifier model selection (Figure 2). We then evaluate this model-building process by examining the biological relevance of shapes (i.e., examining the subtype-specific tissue shapes and cellular structures that correspond to the best features of the classification model) and testing the classifier prediction performance using independent images. Finally, we compare the shape-based diagnostic model to diagnostic models based on traditional histological image features. We show that Fourier shape-based features (1) are capable of classifying H&E-stained renal tumor histological images, (2) out-perform or complement traditional histological image features used in existing automated systems, and (3) are biologically interpretable.

## Methods

### Image datasets

We perform this study on hematoxylin and eosin (H&E) stained histological RGB image datasets acquired from renal tumor samples of patients. In this study, we use two separately acquired datasets: dataset A and dataset B. Both datasets consist of photomicrographs of deidentified renal tumor specimens, derived from human patients. Research was conducted in compliance with the Helsinki Declaration. Tumor specimens were obtained through protocols approved by the Emory University Institutional Review Board, in which patients provided informed consent for residual tumor tissue to be stored in a university tissue bank. Administrators of the tissue bank provided deidentified tissues and associated clinical data (scrubbed of personal health identifiers), to the investigators of this research project. The IRB protocols pertaining to this research project are Emory IRB00045858/1214-2003 and 255–2002. Refer to Figures 1a-d and Figures 1e-h for samples of images in dataset A and dataset B, respectively. After acquisition at constant magnification, a clinician selected 1600 × 1200-pixel portions from whole-slide images and a pathologist assigned each image to a renal tumor subtype. Dataset A contains 48 images with 12 images of each subtype while dataset B has 55 images including 20 chromophobe (CH), 17 clear cell (CC), 13 papillary (PA), and 5 oncocytoma (ON) subtypes. Dataset B has samples with nuclear grade varying from 1 to 4. In total, we analyze 103 renal tumor H&E images.

Automatic color segmentation of the renal tumor images requires an additional reference dataset. The reference dataset need not be the same tissue type. However, the staining protocol should be the same as that of the renal tumor images. We use an H&E stained dataset of 50 randomly selected ovarian cancer images from the NIH Cancer Genome Atlas (TCGA) repository [22]. We use 1024 × 1024-pixel cropped portions of the original slide images. As references, these images are segmented by an expert user with the aid of a user-interactive system [23]. We then use these color-segmented reference images to automatically segment the renal tumor images as described in the following section.

### Automatic color segmentation

H&E staining of a renal tumor histological image enhances three colors: blue-purple, white, and pink. These colors correspond to specific cellular structures. Basophilic structures containing nucleic acids—ribosome and nuclei—tend to stain blue-purple; eosinophilic intra- and extracellular proteins in cytoplasmic regions tend to stain bright pink; empty spaces the lumen of glands do not stain and tend to be white. In order to isolate shapes corresponding to these cellular structures, we segment the three colors of every image using an automatic color segmentation method [23].

**Figure 3:**
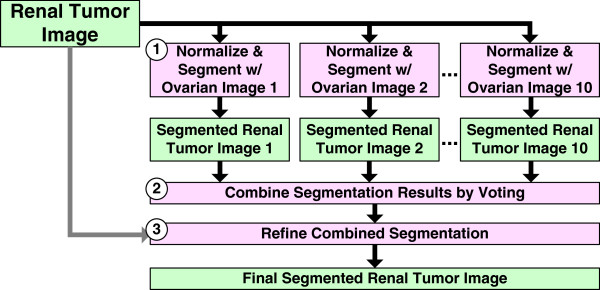
**Renal tumor images are automatically segmented using ten reference ovarian cancer images.** The three main steps of the system are 1) normalization and segmentation using each reference image, 2) combination of segmentation labels by voting, and 3) refinement of combined segmentation by re-classifying pixels in the original color space.

We use two batches of renal tumor images with very different stain colors. Batch-related variation in stain colors is a common problem in histological image analysis. As such, we use a robust automatic color segmentation system (Figure 3). Briefly, our system incorporates knowledge from pre-segmented reference images (the ovarian cancer images) to normalize and segment renal tumor images. In order to make our system robust to the choice of reference image, we normalize and segment each renal tumor image using 10 ovarian cancer reference images (Figure 3, Step 1). We select 10 optimal ovarian cancer reference images from a set of 50 ovarian cancer images using the methodology described by [23]. The segmentation process first normalizes renal tumor image colors to the reference image colors, and then classifies the pixel into one of three groups (nuclei, cytoplasm, or lumen). Pixel classification is performed using a three-class linear discriminant classifier (LDA). We train the classifier using colors and labels in the reference image and classify pixels in the normalized renal tumor images.

The 10 segmentation labels for each pixel (one for each ovarian reference image) are combined using a voting scheme (Figure 3, Step 2). Voting chooses the segmentation label most frequently assigned to a pixel as its preliminary label.

**Figure 4:**
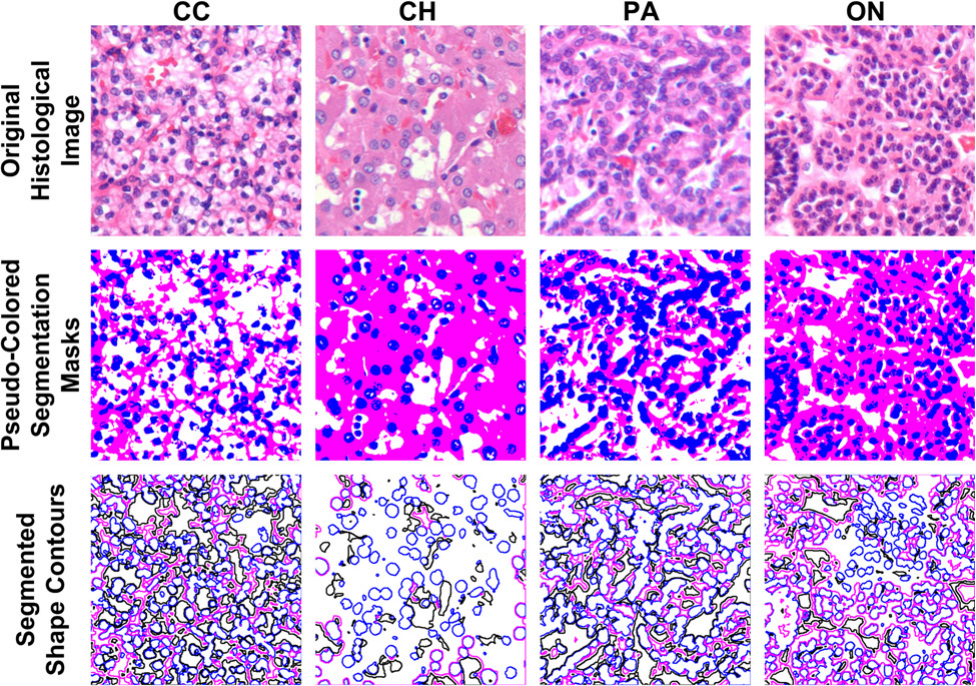
**Color segmentation results and shape contours in three masks for four renal tumor subtypes: clear cell (CC), chromophobe (CH), papillary (PA), and oncocytoma (ON).***First row*: original histological renal tumor subtype images; *second row*: pseudo colored segmentation masks, where blue, white and pink colors correspond to nuclear, cytoplasmic and no-stain/glandular masks, respectively; *third row*: segmented shape contours in nuclear (blue), no-stain/glandular (black), and cytoplasmic (pink) masks.

The preliminary labels obtained by classification and voting are good approximations of the ground truth labels, but we further refine this segmentation using the LDA classifier (Figure 3, Step 3). This step trains the LDA classifier using colors from the original renal tumor image (before normalization) and using preliminary labels. The trained classifier is then used to re-classify all pixels in the renal tumor image. Intuitively, this is a post-processing step that ensures that the color groupings are separable in the original sample image color space, and that any color distortion introduced by normalization is removed. Figure 4 illustrates some color segmentation results. Compared to the ground truth, (expert user-interactive segmentation) the overall segmentation accuracy is greater than 89%.

After segmentation, we extract a binary mask for each stain and apply morphological operations to the binary mask to connect broken boundaries and separate overlapping objects. Namely, we dilate objects in the nuclear mask with a circular structural element with a two-pixel radius and erode objects in the cytoplasmic and glandular masks with a circular structural element with a three-pixel radius. Finally, from all binary masks, we remove small noisy regions with area less than five pixels and extract outer boundaries of the remaining connected objects for further analysis.

### Shape descriptors

We use Fourier shape descriptors to represent shape contours. If we represent each shape contour using parametric equations, (*x*(*t*), *y*(*t*)), the Fourier series expansion for the one-dimensional periodic function *x*(*t*) and *y*(*t*) is given by

$$x\left(t\right)=A_{0}+\sum_{n=1}^{\infty }a_{n}\cos\frac{2\mathrm{n\pi t}}{T}+b_{n}\sin\frac{2\mathrm{n\pi t}}{T}$$

$$y\left(t\right)=C_{0}+\sum_{n=1}^{\infty }c_{n}\cos\frac{2\mathrm{n\pi t}}{T}+d_{n}\sin\frac{2\mathrm{n\pi t}}{T}$$

where *n* is the number of harmonics. We estimate the Fourier coefficients *A*_*0*_, *C*_*0*_, *a*_*n*_, *b*_*n*_*, c*_*n*_, and *d*_*n*_ by the formulas illustrated in [14]. *A*_*0*_ and *C*_*0*_ correspond to the location of a shape, so we do not consider them as shape descriptors. *a*_*n*_, *b*_*n*_*, c*_*n*_, and *d*_*n*_ are the shape descriptors that have commonly been used for shape discrimination [16,24] and shape retrieval [15,25] applications in 4*N dimensional space, where *N* is the number of harmonics. However, we are classifying images based on the distribution of multiple shapes within the images and not based on individual shapes. Therefore, we quantify the distributions of an individual descriptor over all the shapes in an image mask and use these distributions as shape-based features for classification (described in the next section). The distribution of four coefficients, *a*_*n*_, *b*_*n*_*, c*_*n*_, *d*_*n*_, for harmonic *n* cannot be used separately because they jointly describe an ellipse:

$$x_{n}\left(\theta \right)=a_{n}\cos\theta +b_{n}\sin\theta$$

$$y_{n}\left(\theta \right)=c_{n}\cos\theta +d_{n}\sin\theta$$

where

$$\theta =\frac{2\mathrm{n\pi t}}{T}$$

However, using both the semi-major and semi-minor axis lengths of ellipses, we can capture the shape patterns. We quantify semi-major and semi-minor axis lengths as follows. The magnitude of the ellipse phasor is given by

(1) $$r\left(\theta \right)=\sqrt{x_{n}^{2}+y_{n}^{2}}$$

We can locate the extrema of this phasor magnitude by differentiating equation (1) and solving for its root. The resulting solution for *θ* is

(2) $$\theta_{n}=\frac{1}{2}\tan^{-1}\left[\frac{2\left(a_{n}b_{n}+c_{n}d_{n}\right)}{a_{n}^{2}+c_{n}^{2}-b_{n}^{2}-d_{n}^{2}}\right],\mathrm{where}\;0\leq \theta \leq \pi$$

Now, as *r*(*θ*) describes an ellipse, *θ*_*n*_ gives the location of either the major or minor axis while the other axis is given by θn+π2. Therefore, semi-major and semi-minor axes are given by

(3) $$r_{n}^{1}=\max\left(r\left(\theta_{n}\right),r\left(\theta_{n}+\frac{\pi }{2}\right)\right)$$

and

(4) $$r_{n}^{2}=\min\left(r\left(\theta_{n}\right),r\left(\theta_{n}+\frac{\pi }{2}\right)\right)$$

$$r_{n}^{1}$$

and $r_{n}^{2}$ capture the magnitude of a shape’s variation in the *n*^th^ harmonic. For *n* = 1, $r_{n}^{1}$ and $r_{n}^{2}$ encode the size of the shape. For *n* > 1, $r_{n}^{1}$ and $r_{n}^{2}$ encode the complexity of the shape. For simpler shapes, i.e. closer to an ellipse, $r_{n}^{1}$ and $r_{n}^{2}$ quickly reduce to zero, with increasing *n,* while for more complex shapes, they reduce slowly. Therefore, $r_{n}^{1}$ and $r_{n}^{2}$ approximately describe a shape and its complexity (similar to the original Fourier coefficients: *a*_*n*_-*d*_*n*_), but can be separated while quantifying the amount of variation in a particular harmonic. Therefore, instead of using individual descriptors, we use the semi-major (greater of $r_{n}^{1}$ or $r_{n}^{2}$) and semi-minor axis lengths as our shape descriptors. For quantifying shapes, we capture information using up to 10 harmonics to determine how many harmonics are sufficient for image representation and subtype classification.

**Figure 5:**
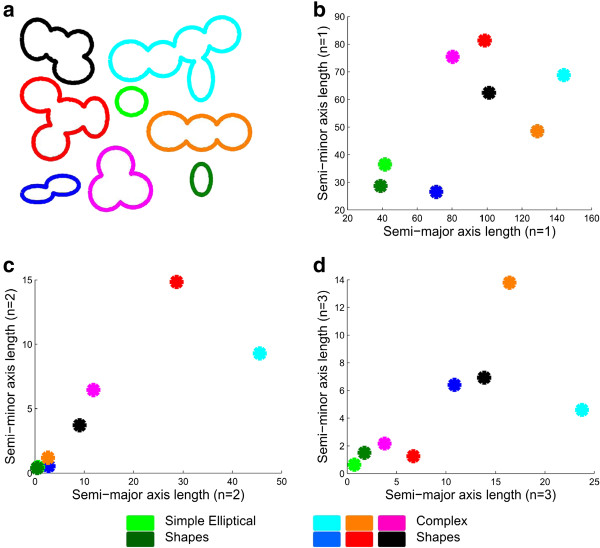
**Axis lengths of shape descriptors capture the complexity of shapes in synthetic images. a**) We use several synthetic shapes to illustrate the utility of Fourier shape descriptors in capturing shape complexity. The green and light green shapes are the simplest elliptical shapes. **b**-**d**) Major and minor axis lengths (in pixels) of the Fourier descriptor ellipses in (**a**), for harmonics n = 1, 2 and 3. Marker colors in (**b**-**d**) correspond to shape colors in (**a**). For first harmonic (*n* = 1), axis lengths represent size and eccentricity of the shape. For *n* > 1, axis lengths represent the detail or complexity of the shape. Therefore, simple green shapes (closer to an ellipse) have small axis lengths, while other complex shapes have larger axis lengths.

Figure 5 illustrates shape axes descriptors for synthetically generated clusters of nuclei. In Figure 5b, for the 1st harmonic, axes features describe size and eccentricity of a shape. For higher harmonics axis lengths encode detail about the shape. Therefore, in Figure 5c and Figure 5d, for the 2nd and 3rd harmonics, simple (closer to an ellipse) shapes (such as the green shapes) have axis lengths close to zero while all other shapes have larger axis lengths.

**Figure 6:**
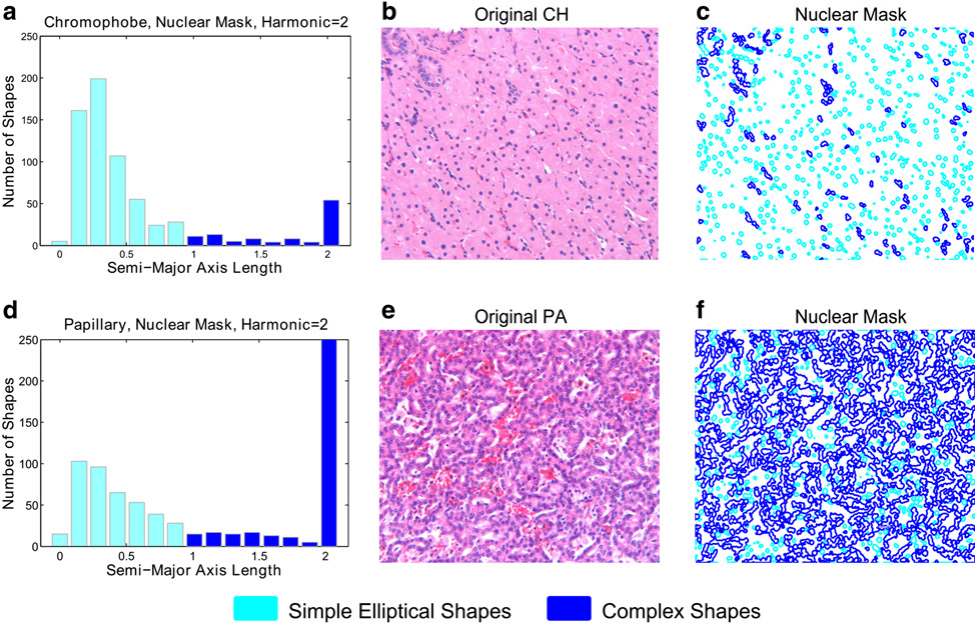
**Fourier shape features discriminate simple and complex shapes in histological renal tumor images.** The bar graphs illustrate the distribution of the second harmonic’s major axis length of all the shapes in the nuclear mask for (**a**) a chromophobe and (**d**) a papillary image. (**b**) - (**c**) and (**e**) - (**f**) are original image and nuclear mask shapes of chromophobe and papillary, respectively. *Cyan shapes*: simple elliptical nuclei for which the 2nd harmonic major axis length, representing amount of detail, falls in the first seven bins of the histogram (cyan bars in the bar graph); *Blue shapes*: complex nuclear clusters for which the 2nd harmonic major axis length falls in the last seven bins of the histogram (blue bars in the bar graph). It can be observed that, due to the complex clusters of nuclei, papillary has more shapes that have high major axis lengths. Therefore, the frequency of shapes in these bins can be an informative feature for distinguishing papillary from chromophobe.

Figure 6 illustrates the ability of the axis length distribution to capture the shape profile of an image. In this figure, we are considering nuclear (blue) mask shapes for two RCC subtypes: chromophobe and papillary. Figures 6a and d represent the distribution of major axis length at harmonic two in the shapes of the images in Figures 6b and e, respectively. The second harmonic captures the complexity of the shape approximation. Although these histograms do not capture the spatial positions of shapes in histopathological images, spatial positions are not useful because the positions of objects (e.g., nuclei) in histopathological images are highly variable from image to image. Instead, these histograms capture the overall proportion of complex or simple shapes in a histopathological image. Thus, for complex shapes like papillary nuclear clusters (resulting from overlapping nuclei in histology), the major axis length of the second harmonic tends to have higher values compared to that of simpler shapes like individual circular nuclei. Consequently, the distribution of shape major axis lengths in papillary images is different from that of chromophobe images. In Figure 6c and Figure 6f corresponding to the histograms in Figure 6a and Figure 6d, respectively we have outlined, in cyan, shapes with values of major axis length that fall in the lower seven bins. Shapes with values of major axis length falling in the upper eight bins are outlined in blue. We can observe that the chromophobe image (Figures 6a, b and c) has a dominant pattern of simple shapes as compared to the papillary image (Figures 6d, e and f). As described in the next section, discretization of axis lengths of all shapes in an image is the basis for representing a histological image as a multi-feature observation.

### Discretization of shape descriptors

In order to develop a classification system, we represent each image as a single observation with a fixed number of features. Due to the variable number of shapes in each image, we quantify the distribution of shape descriptors (axis lengths) to create a “shape profile”, represented as a histogram. We determine the dynamic range of each histogram by computing interquartile distances of shape descriptor distributions from the training set. Interquartile distance is the distance between the 25th and 75th percentiles of a distribution [26]. Mathematically, $R_{n}^{c,m}$ is the distribution of axis lengths over all shapes in all images in the training dataset for a particular combination of harmonic (*n*), axis type (*c*) and mask (*m*). Let function *f*_*P*_ (*R*) return the *p*^*th*^ percentile of distribution *R*, then the interquartile distance (IQD) is given by

(5) $$\mathrm{IQD}\left(R\right)=f_{0.75}\left(R\right)-f_{0.25}\left(R\right)$$

**Figure 7:**
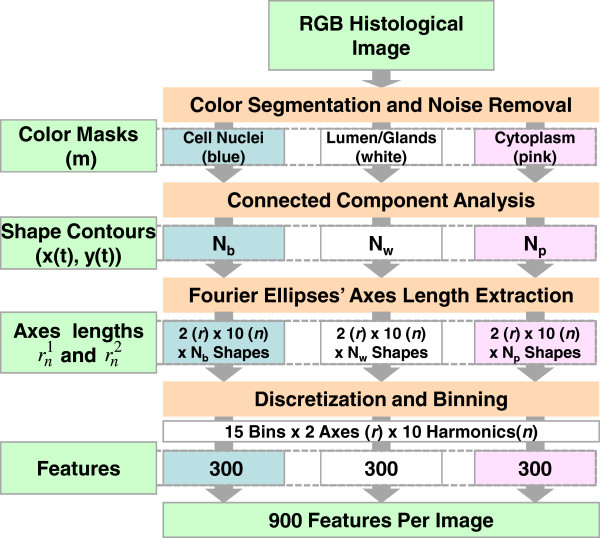
**The data flow for extraction of 900 shape-based features from a histological image.** First, we segment the RGB histological image based on stains: blue (nuclei), pink (cytoplasm), and white (no-stain/gland). Then based on segmented results, we generate three binary masks corresponding to three stains (blue:b, white:w, pink:p). For each mask, we obtain the contour for all shapes after noise filtering using connected component analysis. N_m_ is number of shapes in *m* mask, where *m* ∈ {*b*, *w*, *p*}. We then extract shape axes descriptors (2 axes*10 harmonics) for each shape contour and bin them to produce 2*10 histograms for each mask (3 masks*10*2 histograms in an image). Due to the variation in dynamic range of the two axes and harmonics, we use data-dependent histogram ranges with 15 bins per histogram. We use the histogram frequencies as features for our image classification.

**Figure 8:**
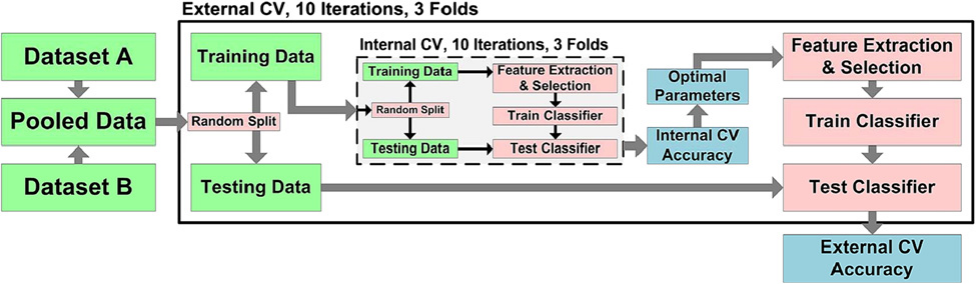
**Evaluation of classification performance using nested cross-validation (CV).** Internal cross-validation (CV) estimates optimal classifier model parameters over three folds and 10 iterations. The parameters optimized include SVM kernel, SVM cost, number of features and number of harmonics. External CV evaluates the optimal model by classifying independent samples.

Using equation (5), we $R_{n}^{c,m}$:

(6) $$L_{n}^{c,m}=\max\left(0,\left(f_{0.5}\left(R_{n}^{c,m}\right)-2*\mathrm{IQD}\left(R_{n}^{c,m}\right)\right)\right)$$

(7) $$U_{n}^{c,m}=f_{0.5}\left(R_{n}^{c,m}\right)+2*\mathrm{IQD}\left(R_{n}^{c,m}\right)$$

where *L*, *U* are the lower and upper bounds of the range, respectively. Outliers bin into the edges of the histogram and may be informative features. Axis lengths are always positive, therefore the lower bound of the range is forced to be greater than or equal to zero. Figure 7 illustrates the data flow from a histological RGB image to a list of 900 features. The procedure is as follows:

1. Generate a binary mask for each color in the histological image. We use three colors for H&E stained RCC images: blue (nuclear), white (no-stain/glandular), and pink (cytoplasmic).

2. Extract contours for all shapes in a mask after connected component analysis.

3. Extract axis lengths for Fourier ellipses ($r_{n}^{1}$ and $r_{n}^{2}$) for the first 10 harmonics (n). This will give us 2*10 variables for each shape.

4. For each harmonic (n), axis type (c), and mask (m), perform a binning procedure (Figure 8). We generate 20 histograms for each mask. We use 15 bins and a range determined by $L_{n}^{c,m}$ and $U_{n}^{c,m}$ as previously described.

5. Combine histogram frequency from the three masks to generate a list of 900 shape-based features

There are a number of advantages in using discretization rather than Euclidian distance to compare images. First, the axes of shapes that are similar, but perhaps not identical, fall into the same histogram bin. Similar histogram frequencies can be interpreted as a similarity of shapes between images. Second, bins sensitive to noise or outlier shapes in any sample will be rejected during feature selection. Finally, discriminating features can be components corresponding to multiple types of shapes rather than components corresponding to the most prominent characteristic shape.

### Traditional features

Traditional features in computer-aided diagnosis include texture, morphological, topological, and nuclear. In order to compare shape-based features to these traditional features, we extract additional features from histological renal tumor images.

For texture, we have two sets of features: Gray-Level Co-occurrence Matrix (GLCM) and wavelet. For GLCM features, we extract a 16 × 16 GLCM matrix for each gray-scale tissue image with 16 quantization levels [27]. Using this matrix, we extract 13 texture properties including contrast, correlation, energy (angular second moment), entropy, homogeneity (inverse difference moment), variance, sum average, sum variance, sum entropy, difference variance, difference entropy, and two information measures for correlation. These features are reported to successfully capture texture properties of the image and are very useful in automated cancer grading [12,27,28].

For wavelet features, we perform three-level wavelet (db6) packet decomposition [29] of the gray-level tissue image and extract energy and entropy [30] of 84 coefficient matrices (level 1, 2 and 3), producing 168 features. Wavelet features capture texture properties of an image.

For morphological features, we use color-GLCM, a method proposed by Chaudry et al. to classify renal tumor subtypes. This method generates a four-level gray-scale image from four color stains in H&E-stained images [7]. The four colors resulting from H&E-stained images (blue, white, pink, and red) correspond to segmented regions of nuclei, lumen, cytoplasm, and red blood cells. We then extract a 4 × 4 GLCM matrix for the gray-scale image. We extract 21 features from this matrix including 16 elements of the 4 × 4 GLCM matrix, contrast, correlation, energy (angular second moment), entropy, and homogeneity (inverse difference moment). These features capture morphological features of the image such as stain area and stain co-occurrence properties.

For topological features, we use a graph-based method. Several researchers have proposed graph-based features to capture the distribution of patterns in an image. Biologically, these features capture the amount of differentiation (related to cancer grade) in a histological image. We morphologically erode our nuclear mask to separate nuclear clusters and use their centroids (nuclear centers) for this analysis. First, we create a Voronoi diagram from these centers and then calculate area and perimeter of each region and all side-lengths. We then calculate mean, minimum, maximum, and disorder of the distribution to produce 12 features [12]. The disorder, D, of a distribution, r, is given by Dr=1−1+σrμr−1, where σ_*r*_ and *μ*_*r*_ are standard deviation and mean of r, respectively [31]. Second, we calculate the area and side lengths of the Delaunay triangles and extract statistics similar to those of the Voronoi diagram to produce eight more features. Last, we calculate side lengths of the minimum spanning tree and extract the same statistics to produce four more features. In total, we extract 24 topological features.

For nuclear features, we extract nuclear count and elliptical-shape properties, which have proven to be useful for renal carcinoma subtyping and grading [32]. For segmenting nuclear clusters, we use an edge-based method with three steps: concavity detection, straight-line segmentation, and ellipse fitting [33]. We describe each elliptical nucleus using area, major-axis length, minor-axis length, and eccentricity. We then calculate mean, minimum, maximum and disorder of the distribution of these descriptors to produce 16 features. In total, including nuclear count, we extract 17 nuclear features.

We combine the GLCM (13 features), color-GLCM (21), wavelet (168), topological (24), and nuclear (17) features to produce a set of 243 “Combined Traditional” features. Finally, we combinethe “Combined Traditional” (243) and “Shape” (900) features to a produce a set of 1143 “All” features.

### Feature selection and classification

For validation, we combine datasets A and B, then randomly split them into two new training and testing datasets with balanced sampling from both datasets. We perform a three-fold split, in which two folds form the training set while one fold forms the testing set. Each fold acts as a testing set once, resulting in three training–testing sets. We perform 10 iterations of this split to estimate the variance in performance. Thus, there are 30 training–testing sets in the external cross-validation (CV) that produces the final classification accuracy. For each of the 30 training sets, we perform an additional three-fold, 10 iterations of CV to choose an optimal set of classifier and feature selection parameters. This forms the internal CV of a nested CV (Figure 8).

**Figure 9:**
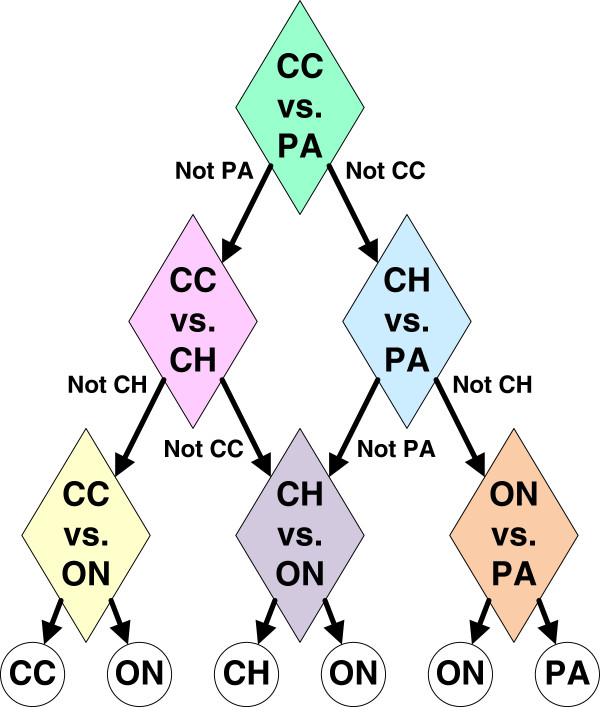
**A multi-class hierarchy of binary renal tumor subtype classifiers, also known as a directed acyclic graph (DAG) classifier.** The overall accuracy of the DAG classifier can be optimized by independently optimizing each binary comparison.

We construct a multi-class classification system consisting of a hierarchy of binary classifiers CC vs. PA, CC vs. CH, CC vs. ON, CH vs. PA, CH vs. ON, and ON vs. PA also called a directed acyclic graph (DAG) classifier [34]. According to Platt et al., the order of binary comparisons has little effect on the overall classification accuracy. Thus, we use the hierarchy illustrated in Figure 9. Each node in the hierarchy is independently optimized such that, for each binary comparison, we choose a set of model parameters (i.e., classifier as well as feature selection parameters). We consider 224 SVM classifier models including 14 kernel types (linear or radial with the gamma parameter ranging from 2^2^, 2^1^, 2^0^to 2^-10^) and 16 cost values (2^-5^, 2^-4^, 2^-3^ to 2^10^) [35,36]. We considered the following feature sizes for different features (e.g., starting feature size:feature step size:ending feature size):

1. GLCM (1:1:13)

2. Color-GLCM (1:1:21)

3. Wavelet (1:5:166)

4. Topological (1:1:24)

5. Nuclear (1:1:17)

6. Combined Traditional (1:6:243)

7. Shape and All (5:5:180)

We choose the feature size step such that the total number of feature sizes is approximately 40. For Shape and All features we also consider number of harmonics (*n* = 2 to 10) as a feature selection parameter. We choose the simplest model with a CV accuracy within one standard deviation of the best performing model [37]. In choosing the simplest model, we give preference to the linear SVM kernel over the radial SVM kernel and lower values of gamma for the radial SVM kernel, SVM cost, number of harmonics, and feature size.

We select features using a feature ranking technique called mRMR (Minimum Redundancy Maximum relevance) [38]. MRMR selects a set of features that maximizes mutual information between class labels and each feature in the set; and minimizes mutual information between all pairs of features in the set. Our features are continuous and, as suggested by Ding et al., we use Mutual Information Quotient (MIQ) optimization after discretization using the following transform:

$$k^{'}=\left\{\begin{array}{c} -1\\ 0\\ 1 \end{array}\begin{array}{c} k<\mu_{k}-\sigma_{k}/2\\ \mu_{k}-\sigma_{k}/2\leq k\leq \mu_{k}+\sigma_{k}/2\\ k>\mu_{k}+\sigma_{k}/2 \end{array}\right\}$$

where *k’* is the transformed feature *k*, *μ*_*k*_ and *σ*_*k*_ are the mean and standard deviation of feature *k* over all samples in the training dataset, respectively.

## Results and discussion

### Shape-based features discriminate renal tumor histological images

Fourier shape-based features are capable of classifying histological renal tumor subtype images with high accuracy and simple classification models. Table 1 lists the shape-based prediction performance of the multi-class renal tumor classifier (using a Directed Acyclic Graph, DAG, classifier [34]) as well as that of each binary comparison (discrimination of every pair of subtypes). The shape-based multi-class classifier predicts the subtypes of renal tumor images with an average accuracy of 77%. The average prediction accuracy for each binary comparison ranges between 83%-96%. Moreover, the classification model for each binary comparison is fairly simple, i.e., each model uses (1) shapes described by lower harmonics, (2) small feature size, and (3) a linear SVM with low cost (Table 2). Refer to Additional file 1 for detailed classifier model selection results.

**Table 1:** Predictive performance of shape-based features

**Endpoint**	**Inner CV accuracy**	**External CV accuracy**
DAG	N/A	0.77 ± 0.03
CH vs. CC	0.83 ± 0.03	0.83 ± 0.05
CH vs. ON	0.83 ± 0.02	0.84 ± 0.04
CH vs. PA	0.97 ± 0.01	0.96 ± 0.02
CC vs. ON	0.90 ± 0.02	0.90 ± 0.07
CC vs. PA	0.96 ± 0.01	0.95 ± 0.04
ON vs. PA	0.94 ± 0.01	0.93 ± 0.04

**Table 2:** Frequently selected model parameters for each binary comparison

**Binary endpoint**	**Harmonic**	**Feature size**	SVM cost*	**SVM gamma**
CH vs. CC	2	15	−1	Linear
CH vs. ON	2	10	1	Linear
CH vs. PA	2	5	−1	Linear
CC vs. ON	2	10	0	Linear
CC vs. PA	2	10	−3	Linear
ON vs. PA	4	5	−1	Linear

* Log_2_values.

**Figure 10:**
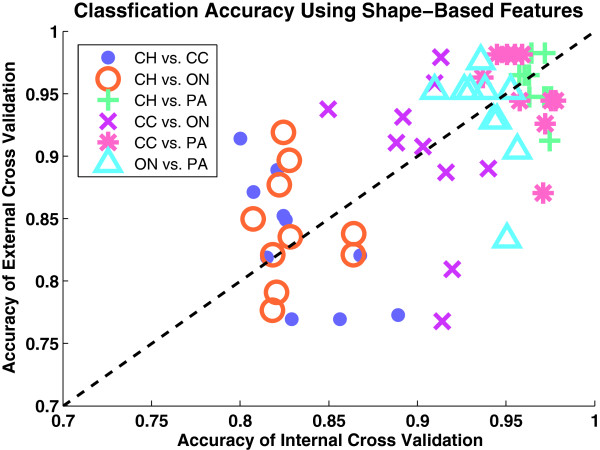
**Cross-validation estimates the prediction performance of shape-based classification models on independent samples.** Scatter plot of inner CV vs. external CV average validation accuracy values over 10 external CV iterations for six pair-wise renal tumor subtype comparisons: CH vs. CC, CH vs. ON, CH vs. PA, CC vs. ON, CC vs. PA, and ON vs. PA. The plotted performance value for each iteration is the average performance over three folds (for external CV) or over 10 iterations and three folds (for internal CV). The optimal classifier model parameters (one set for each point) are selected in the inner CV from a possible set of 72576 models consisting of 36 feature sizes, 14 types of classifiers (linear SVM and radial basis SVM classifiers with 13 different gammas), 16 cost values and 9 harmonic numbers.

We use nested cross-validation (CV) to select prediction model parameters and to evaluate these prediction models on independent data. The nested CV procedure includes 10 iterations of three-fold external CV with 10 iterations of three-fold internal CV. Although there is some variance across the iterations of CV, Figure 10 shows that mean internal CV is a good estimate of mean external CV for each of the binary comparisons. Each point in Figure 10 corresponds to an iteration of external CV for each binary comparison. The horizontal position of each point is internal CV accuracy averaged over 10 iterations and three folds. The vertical position of each point is external CV accuracy averaged over three folds. Classifier model parameters for each point are selected from among 72,576 models consisting of 36 feature sizes, 14 types of SVM classifiers (linear SVM and radial basis SVM classifiers over 13 different gammas), 16 SVM cost values, and 9 values for the number of harmonics. The optimal parameter set for each classifier model corresponds to the simplest model (i.e., smallest feature size, smallest cost, smallest gamma, and smallest number of harmonics) within one standard deviation of the best performing model. This high concordance of internal CV and external CV performance indicates that internal CV performance is predictive of external CV performance and classifier models generated from shape features are robust and will perform similarly for future samples. Moreover, the binary comparisons discriminating CH vs. PA, CC vs. ON, CC vs. PA, and ON vs. PA tend to result in high performance (> 90%) while the binary comparisons discriminating CH vs. CC and CH vs. ON result in moderate performance (~83-84%). We describe the reasons for these observations below.

CC and PA are the most prevalent subtypes of RCC and are generally the easiest for pathologists to visually identify. Consequently, discriminating shape-based features for these classes are easy to identify, resulting in high classification performance. One exception, however, is the CH vs. CC comparison. CH is known to exhibit some CC properties such as clear cytoplasm. As a result, the prominent feature for the CC subtype is sometimes not sufficient for accurate classification of CC and CH. Moreover, the ON renal tumor subtype is histologically and genetically very similar to the CH RCC subtype, despite the fact that ON is a benign tumor whereas CH is a carcinoma [39]. This similarity explains the moderate performance of the CH and ON binary classifier.

### Shape-based features out-perform or complement traditional histological features

**Figure 11:**
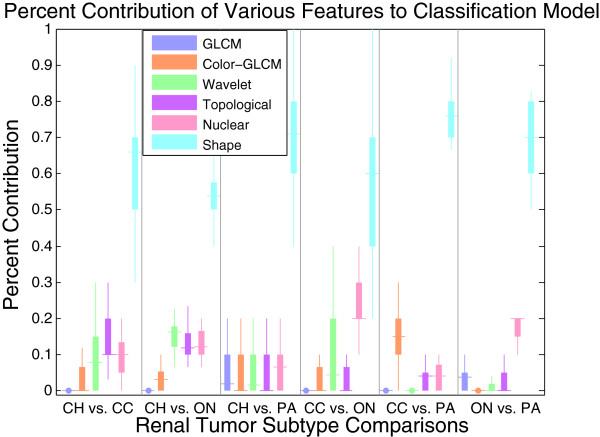
**Renal tumor binary classification models use a variety of features to quantify important biological properties.** Percentage contribution of different features for each binary comparison in ‘All’ features model. The contribution of shape features tends to be greater than 55% for all endpoints (median value, marked by horizontal line).

Table 3 shows that, in comparison to five traditional feature sets, classification of renal tumor subtypes based on shape-based features performs well. In fact, the performance of shape features is similar to the combined traditional features, which includes texture, topological and nuclear properties. In some cases, combining shape-based features with traditional features (i.e., ‘All’ features) improves prediction performance, indicating that shape-based features can complement traditional features. Table 3 lists the means and standard deviations over 10 iterations of external CV for each binary comparison as well as for the multi-class DAG classifier. Figure 11 shows the contribution of each feature type to the classification model when considering ‘All’ features. The box plots in Figure 11 represent the distribution of percent contribution of each feature type to a binary classifier over 10 iterations of external CV. We can make the following observations from Figure 11: 1) Shape features have a high (>55%) contribution for all binary endpoints, which indicates that the feature selection method ranks shape features higher than other features. The contribution is comparatively lower for CH vs. CC, CH vs. ON, and CC vs. ON endpoints because other traditional features were also useful for these endpoints. 2) Nuclear features, which capture nuclear-shape properties, highly contribute to all six endpoints 2) In addition to shape features for the CH vs. ON endpoint, topological, nuclear and wavelet features also contribute to the prediction models, resulting in a 4% increase in accuracy compared to shape features alone. This indicates that, in addition to shape (Fourier and nuclear) properties, CH and ON differ in topological and wavelet properties. 3) Color GLCM performs very well for CC vs. PA classification. Thus, color GLCM is a major contributor for CC vs. PA classification, resulting in a 2% increase in accuracy.

**Table 3:** Classification accuracy of features in external CV*

**Endpoint**	**GLCM**	**Color GLCM**	**Wavelet**	**Topological**	**Nuclear**	**Combined traditional**	**Shape (Proposed)**	**All**
DAG	0.57 ± 0.04	0.67 ± 0.02	0.52 ± 0.06	0.50 ± 0.03	0.66 ± 0.03	0.79 ± 0.04^a^	0.77 ± 0.03^b^	0.78 ± 0.03^c^
CH vs. CC	0.75 ± 0.05	0.77 ± 0.05	0.74 ± 0.05	0.74 ± 0.05	0.76 ± 0.06	0.81 ± 0.03	0.83 ± 0.05	0.82 ± 0.05
CH vs. ON	0.76 ± 0.05	0.68 ± 0.06	0.67 ± 0.05	0.72 ± 0.05	0.79 ± 0.05	0.86 ± 0.05	0.84 ± 0.04	0.88 ± 0.04
CH vs. PA	0.85 ± 0.04	0.95 ± 0.02	0.86 ± 0.05	0.80 ± 0.04	0.91 ± 0.04	0.94 ± 0.02	0.96 ± 0.02	0.96 ± 0.03
CC vs. ON	0.74 ± 0.06	0.78 ± 0.06	0.63 ± 0.03	0.77 ± 0.07	0.93 ± 0.04	0.93 ± 0.04	0.90 ± 0.07	0.91 ± 0.05
CC vs. PA	0.78 ± 0.06	0.97 ± 0.04	0.69 ± 0.09	0.59 ± 0.07	0.76 ± 0.07	0.95 ± 0.05	0.95 ± 0.04	0.97 ± 0.03
ON vs. PA	0.74 ± 0.07	0.86 ± 0.06	0.74 ± 0.07	0.65 ± 0.04	0.96 ± 0.03	0.97 ± 0.03	0.93 ± 0.04	0.92 ± 0.04

* The difference between a, b, and c is not statistically significant; p-values of the null hypothesis between a & b; a & c; and b & c using a t-test are 0.16, 0.70, and 0.23, respectively.

### Shape-based features are biologically interpretable

**Figure 12:**
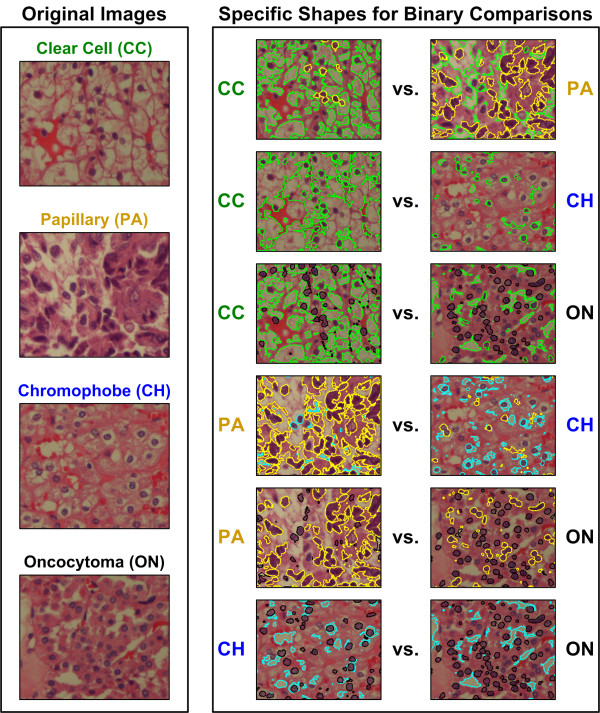
**The top discriminating shapes for six binary endpoints correspond to pathologically significant shapes in histological renal tumor images.** We identify the top 25 features selected for each binary comparison and highlight all shapes in the images that have any Fourier shape-descriptor axes lengths corresponding to these top features. We selectively color the shapes based on “over expression” or increased relative frequency for particular subtypes. *Green shapes*: occur more frequently in clear cell; *yellow shapes*: occur more frequently in papillary; *blue shapes*: occur more frequently in chromophobe; and *black shapes*: occur more frequently in oncocytoma.

Figure 12 illustrates the biological interpretability of shape-based features for each renal tumor subtype. In order to visualize the biological significance of the features identified by our feature selection method, we overlay the top discriminating shapes on the images of renal tumor subtypes for each binary comparison. Feature selection identifies individual shape axes and not entire shapes. Thus, discriminating shapes are shapes with axes values that have been discretized into a bin corresponding to a highly ranked feature. For each binary comparison, we identify all shapes in an image that have Fourier axes values corresponding to the top 25 features. These shapes are selected using features from all images. We set the “number of harmonics” parameter equal to the most selected value during the cross-validation (Table 2). We selectively color the shapes based on “over expression”, or increased relative frequency for particular subtypes. Shapes highlighted in green occur more frequently in CC; yellow shapes occur more frequently in PA; blue shapes occur more frequently in CH; and black shapes occur more frequently in ON. We interpret the biological significance of highlighted shapes for each binary comparison.

Histopathological features of the CC subtype include clear cytoplasm, compact alveolar, tubular, and cystic architecture leading to distinct cell membranes [3]. Comparing CC to PA and ON, we see that clear cytoplasm (no-stain/glandular (white) mask region, outlined with green) is the primary distinguishing characteristic that is noticeably less frequent in PA and ON. On the other hand, because CH images tend to also exhibit halos resembling clear cytoplasm, the distinguishing features between CC and CH are distinct cell membranes (small cytoplasmic (pink) mask areas outlined with green between larger no-stain/glandular (white) mask areas) that are more frequent in CC compared to CH. Similarity in halos and clear cytoplasm shapes is possibly the reason for low accuracy in the CH vs. CC binary classification.

Features of the PA subtype include scanty eosinophilic cytoplasm and a papillary (i.e., finger-like) pattern of growth resulting in long, complex clusters of nuclei [3]. In all comparisons with the PA subtype, complex clusters of nuclei are the dominant distinguishing feature and are generally more prominent in PA (nuclear (blue) mask areas outlined with yellow). The frequency of nuclear shapes in ON appears to be similar to that of PA. However, the nuclear clusters in PA are generally larger and more irregular due to the clustering, resulting in different Fourier shape axes values.

Histopathological features of the CH subtype include wrinkled nuclei with perinuclear halos [3]. When comparing CH to PA or ON, our feature extraction and selection method identifies these halos (no-stain/glandular (white) mask areas, outlined with blue). In addition, single nuclei become dominant when comparing CH to PA.

Histopathological features of the ON subtype include granular cytoplasm with round nuclei, usually arranged in compact nests or microcysts [3]. These round nuclei appear to be dominant in ON when compared to other subtypes. It can be observed that dominant features for both CH and ON are present in the opposite subtype as well. Hence, the difficulty in distinguishing the two subtypes.

### Limitations and computational complexity of shape-based features

Some limitations of shape-based features for histological image classification depend on the specific biological application. Shape-based features may not be suitable for cases in which the primary discriminating features are not based on shapes. For example, in cancer grading applications, topological and texture properties may be more useful than shape-based features. Moreover, as we have seen the results of Table 3 and Figure 11, shape-based features may not capture all of the important distinguishing information. For example, in the case of the CH vs. ON endpoint, the addition of texture and wavelet features to shape-based features increases prediction performance by 4%. In addition, for the CC vs. PA endpoint, inclusion of the GLCM texture features increases prediction performance by 2%. Thus, shape-based features are limited to clinical prediction applications that are inherently shape-based, but, in such cases, may be used to complement other non-shape-based features.

The computational complexity of shape-based features is higher than those of traditional histological feature extraction and analysis methods, but should not prevent implementation in a clinical setting. To convert a RGB histological image (1600x1200 pixel portions) into 900 shape-based features (Figure 7), a desktop computer (Intel Xeon E5405 quad-core processor, 20 GB RAM) requires an average of 74.96 seconds. Compared to some histological image features, this processing time is high. However, the processing time depends on the number of harmonics used for representation and the number of shapes in an image. We have reported the processing time for extracting features from the first ten harmonics. However, in practice, we have observed that all optimized models use less than five harmonics. Optimization of these parameters to identify a predictive model can be time consuming depending on the size of the training set. However, in a clinical setting, such a model would only need to be optimized once, and then periodically updated with new patient data. In a clinical scenario, a pathologist that requires a histological diagnosis for a patient would submit a few image samples from a tissue biopsy to a pre-optimized prediction system. Computational time for processing and predicting based on these image samples would be negligible compared to time required for biopsy, image acquisition, and consultation with a pathologist.

## Conclusions

We presented a novel methodology for automatic clinical prediction of renal tumor subtypes using shape-based features. These shape-based features describe the distribution of shapes extracted from three dominant H&E stain colors in renal tumor histopathological images. We evaluated the four-class prediction performance of shape-based classification models using 10 iterations of three-fold nested CV. The overall classification accuracy of 77% (average external CV accuracy) is favorable compared to previous methods that use traditional textural, morphological, and wavelet-based features. Moreover, results indicate that combining shape-based features with traditional histological image features can improve prediction performance. The biological significance of the characteristic shapes identified by our algorithm suggests that this automatic diagnostic system mimics the diagnostic criteria of pathologists. We applied this methodology to renal tumor subtype prediction. However, the methodology may be extended to any histological image classification problem that traditionally depends on visual shape analysis by a pathologist. Moreover, these shape-based features may be coupled with other image features to achieve higher diagnostic accuracy.

## Abbreviations

RCC: Renal cell carcinoma; CC: Clear cell; PA: Papillary; CH: Chromophobe; ON: Oncocytoma; mRMR: Minimum redundancy maximum relevance; DAG: Directed acyclic graph; CV: Cross-validation; GLCM: Gray-level co-occurrence matrix; DAG: Directed acyclic graph; LDA: Linear discriminant analysis; SVM: Support vector machine.

## Competing interests

The authors declare that they have no competing interests.

## Authors’ contributions

SK designed the image feature extraction methods (including color segmentation, shape descriptor extraction and discretization), contributed to the design of validation experiments and shape feature visualization, implemented all methods, and drafted the manuscript. JHP designed validation experiments and shape feature visualization, and contributed to the design of feature extraction methods. ANY provided all biological specimens and provided biological interpretation of informative shapes for each tumor subtype. MDW initiated the development of the automatic cancer diagnostic system, acquired funding to sponsor this multi-year effort, and directed the development of the shape-based analysis methodology and publication. All authors read and approved the final manuscript.

## Pre-publication history

The pre-publication history for this paper can be accessed here:

http://www.biomedcentral.com/1471-2342/13/9/prepub

## Supplementary Material

Additional file 1 This file includes figures describing classifier model parameter space investigation. Click here for file

## References

[B1] Siegel R Ward E Brawley O Jemal A Cancer statistics, 2011 CA Cancer J Clin 2011 61 4 212 236 10.3322/caac.20121 21685461

[B2] Teloken PE Thompson RH Tickoo SK Cronin A Savage C Reuter VE Russo P Prognostic Impact of Histological Subtype on Surgically Treated Localized Renal Cell Carcinoma J Urol 2009 182 5 2132 2136 10.1016/j.juro.2009.07.019 19758615 PMC4169873

[B3] Eble J Sauter G Epstein J Sesterhenn I Pathology and genetics of tumours of the urinary system and male genital organs 2004 Lyon: IARC press Lyon

[B4] Demir C Yener B Automated cancer diagnosis based on histopathological images: a systematic survey 2005 Tech Rep: Rensselaer Polytechnic Institute

[B5] Gurcan MN Boucheron LE Can A Madabhushi A Rajpoot NM Yener B Histopathological image analysis: A review Biomed Eng, IEEE Rev 2009 2 147 171 10.1109/RBME.2009.2034865 PMC2910932 20671804

[B6] Chaudry Q Raza SH Sharma Y Young AN Wang MD Improving renal cell carcinoma classification by automatic region of interest selection BioInformatics and BioEngineering, 2008 BIBE 2008 8th IEEE International Conference on: 2008 2008 Athens, Greece: IEEE 1 6 10.1109/BIBE.2008.4696796 PMC5382997 28393153

[B7] Chaudry Q Raza SH Young AN Wang MD Automated Renal Cell Carcinoma Subtype Classification Using Morphological, Textural and Wavelets Based Features J Signal Process Syst 2009 55 1 15 23 10.1007/s11265-008-0214-6 PMC5267341 28133502

[B8] Waheed S Moffitt RA Chaudry Q Young AN Wang MD Computer Aided Histopathological Classification of Cancer Subtypes Bioinformatics and Bioengineering, 2007 BIBE 2007 Proceedings of the 7th IEEE International Conference on: 2007 2007 Boston, United States: IEEE 503 508

[B9] Choi HJ Choi HK Grading of renal cell carcinoma by 3D morphological analysis of cell nuclei Comput Biol Med 2007 37 9 1334 1341 10.1016/j.compbiomed.2006.12.008 17331492

[B10] François C Moreno C Teitelbaum J Bigras G Salmon I Danguy A Brugal G van Velthoven R Kiss R Decaestecker C Improving accuracy in the grading of renal cell carcinoma by combining the quantitative description of chromatin pattern with the quantitative determination of cell kinetic parameters Cytometry B Clin Cytom 2000 42 1 18 26 10.1002/(SICI)1097-0320(20000215)42:1<18::AID-CYTO4>3.0.CO;2-S 10679739

[B11] Raza SH Sharma Y Chaudry Q Young AN Wang MD Automated classification of renal cell carcinoma subtypes using scale invariant feature transform Engineering in Medicine and Biology Society, 2009 EMBC 2009 Annual International Conference of the IEEE: 3–6 Sept. 2009 2009 2009 Minneapolis, United States: IEEE 6687 6690 10.1109/IEMBS.2009.5334009 PMC5003033 19964707

[B12] Doyle S Agner S Madabhushi A Feldman M Tomaszewski J Automated grading of breast cancer histopathology using spectral clustering with textural and architectural image features Biomedical Imaging: From Nano to Macro, 2008 ISBI 2008 5th IEEE International Symposium on: 2008 2008 Paris, France: IEEE 496 499

[B13] Sertel O Kong J Catalyurek UV Lozanski G Saltz JH Gurcan MN Histopathological image analysis using model-based intermediate representations and color texture: Follicular lymphoma grading J Signal ProcessSyst 2009 55 1 169 183 10.1007/s11265-008-0201-y

[B14] Kuhl F Giardina C Elliptic Fourier features of a closed contour Comput Graph Image Process 1982 18 3 236 258 10.1016/0146-664X(82)90034-X

[B15] Lee D Antani S Long L Similarity measurement using polygon curve representation and fourier descriptors for shape-based vertebral image retrieval SPIE Medical Imaging 2003 2003 1283 1291

[B16] Rangayyan R El-Faramawy N Desautels J Alim O Measures of acutance and shape for classification of breast tumors IEEE Trans Med Imaging 1997 16 6 799 810 10.1109/42.650876 9533580

[B17] Cukierski W Nandy K Gudla P Meaburn K Misteli T Foran D Lockett S Ranked retrieval of segmented nuclei for objective assessment of cancer gene repositioning BMC Bioinforma 2012 13 1 232 10.1186/1471-2105-13-232 PMC3484015 22971117

[B18] Yang L Tuzel O Chen W Meer P Salaru G Goodell LA Foran DJ PathMiner: a Web-based tool for computer-assisted diagnostics in pathology IEEE Trans Inf Technol Biomed 2009 13 291 299 19171530 10.1109/TITB.2008.2008801 PMC3683402

[B19] Comaniciu D Meer P Suri JS, Setarehdan SK, Singh S Cell Image Segmentation for Diagnostic Pathology Advanced Algorithmic Approaches to Medical Image Segmentation 2002 London: Springer 541 558

[B20] Lao Z Shen D Xue Z Karacali B Resnick S Davatzikos C Morphological classification of brains via high-dimensional shape transformations and machine learning methods Neuroimage 2004 21 1 46 57 10.1016/j.neuroimage.2003.09.027 14741641

[B21] Berg AC Berg TL Malik J Shape matching and object recognition using low distortion correspondences Computer Vision and Pattern Recognition, 2005 CVPR 2005 IEEE Computer Society Conference on: 2005 2005 2005 San Diego, United States: IEEE 26 33 Volume 21.

[B22] McLendon R Friedman A Bigner D Van Meir E Brat D Mastrogianakis G Olson J Mikkelsen T Lehman N Aldape K Cancer Genome Atlas Research Network. Comprehensive genomic characterization defines human glioblastoma genes and core pathways Nature 2008 455 7216 1061 1068 10.1038/nature07385 18772890 PMC2671642

[B23] Kothari S Phan JH Moffitt RA Stokes TH Hassberger SE Chaudry Q Young AN Wang MD Automatic batch-invariant color segmentation of histological cancer images Biomedical Imaging: From Nano to Macro, 2011 IEEE International Symposium on: March 30 2011-April 2 2011 2011 2011 Chicago, United States: IEEE 657 660 10.1109/ISBI.2011.5872492 PMC4983436 27532016

[B24] Persoon E Fu K Shape discrimination using Fourier descriptors IEEE Trans Syst Man Cybern 1977 7 3 170 179 10.1109/tpami.1986.4767799 21869355

[B25] Wong W Shih F Liu J Shape-based image retrieval using support vector machines, Fourier descriptors and self-organizing maps Inform Sci 2007 177 8 1878 1891 10.1016/j.ins.2006.10.008

[B26] McGill R Tukey J Larsen W Variations of box plots Am Stat 1978 32 1 12 16

[B27] Haralick R Shanmugam K Dinstein I Textural features for image classification IEEE Trans Syst Man Cybern 1973 3 6 610 621

[B28] Tae-Yun K Hyun-Ju C Soon-Joo C Heung-Kook C Study on texture analysis of renal cell carcinoma nuclei based on the Fuhrman grading system Enterprise networking and Computing in Healthcare Industry, 2005 HEALTHCOM 2005 Proceedings of 7th International Workshop on: 2005 2005 384 387

[B29] Laine A Fan J Texture classification by wavelet packet signatures IEEE Trans Pattern Anal Mach Intell 1993 15 11 1186 1191 10.1109/34.244679

[B30] Jafari-Khouzani K Soltanian-Zadeh H Multiwavelet grading of pathological images of prostate IEEE Trans Biomed Eng 2003 50 6 697 704 10.1109/TBME.2003.812194 12814236

[B31] Sudbø J Marcelpoil R Reith A New algorithms based on the Voronoi Diagram applied in a pilot study on normal mucosa and carcinomas Anal Cell Pathol 2000 21 2 71 86 11310643 10.1155/2000/389361 PMC4618427

[B32] Kothari S Phan JH Young AN Wang MD Histological Image Feature Mining Reveals Emergent Diagnostic Properties for Renal Cancer Bioinformatics and Biomedicine (BIBM), 2011 IEEE International Conference on: 2011 2011 Atlanta, United States: IEEE 422 425 10.1109/BIBM.2011.112 PMC5287706 28163980

[B33] Kothari S Chaudry Q Wang MD Extraction of informative cell features by segmentation of densely clustered tissue images Engineering in Medicine and Biology Society, 2009 Annual International Conference of the IEEE: 3–6 Sept. 2009 2009 2009 Minneapolis, United States: IEEE 6706 6709 10.1109/IEMBS.2009.5333810 PMC4983437 19964444

[B34] Platt J Cristianini N Shawe-Taylor J Large margin DAGs for multiclass classification Adv Neural Inf Process Syst 2000 12 3 547 553

[B35] Boser B Guyon I Vapnik Vtraining algorithm for optimal margin classifiers 1992 New York, NY, USA: ACM 144 152

[B36] Chang CC Lin CJ LIBSVM: A library for support vector machines ACM Trans Intell Syst Technol (TIST) 2011 2 3 27

[B37] Hastie T Tibshirani R Friedman JH The elements of statistical learning: data mining, inference, and prediction 2009 Verlag: Springer

[B38] Ding C Peng H Minimum redundancy feature selection from microarray gene expression data J Bioinforma Comput Biol 2005 3 2 185 10.1142/S0219720005001004 15852500

[B39] Sakai Y Watanabe S Matsukuma S Chromophobe renal cell carcinoma showing oncocytoma-like hyalinized and edematous stroma: a case report and review of the literature Urol Oncol 2004 22 6 461 464 10.1016/j.urolonc.2004.03.015 15610861

