# Supplementary material for: Histological image classification using biologically interpretable shape-based features
Source: BMC Med Imaging. 2013 Mar 13;13:9. doi: 10.1186/1471-2342-13-9 (PMC3623732; doi:10.1186/1471-2342-13-9)
Supplement: Additional file 1 — This file includes figures describing classifier model parameter space investigation. [file 1471-2342-13-9-S1.docx]

**Classifier Model Parameter Space Investigation**

**Figure S1** shows that the optimal classifier parameters correspond to fairly simple models. The parameters are optimized by selecting the simplest model with predictive performance within one standard deviation of the highest performing model. We define a simple model as one that has small feature size, low SVM cost, low SVM gamma (or is a linear SVM), and that prefers features from smaller Fourier shape descriptor harmonics. Although we consider ten harmonics of shape-based features, parameter selection usually only selects the first few (< 4) harmonics (**Figure S1a**). In **Figure S1a**, even though the best choice for number of harmonics varies for different binary classifiers, all binary classifiers have a decreasing trend of selecting a high number of harmonics. **Figure S1b** illustrates the distribution of feature sizes selected for binary classifier models. We can observe that there is a decreasing trend of selecting large feature sizes and, in most cases, less than 20 features were selected. **Figure S1c** and **Figure S1d** show that optimal SVM cost and SVM gamma selections are also low, with preference given to linear SVMs over non-linear radial basis SVMs. **Table 2** in the manuscript lists the most frequently selected parameters for each of the six binary classifiers.

**Figure S1. Parameter space investigation for shape-based classification models**


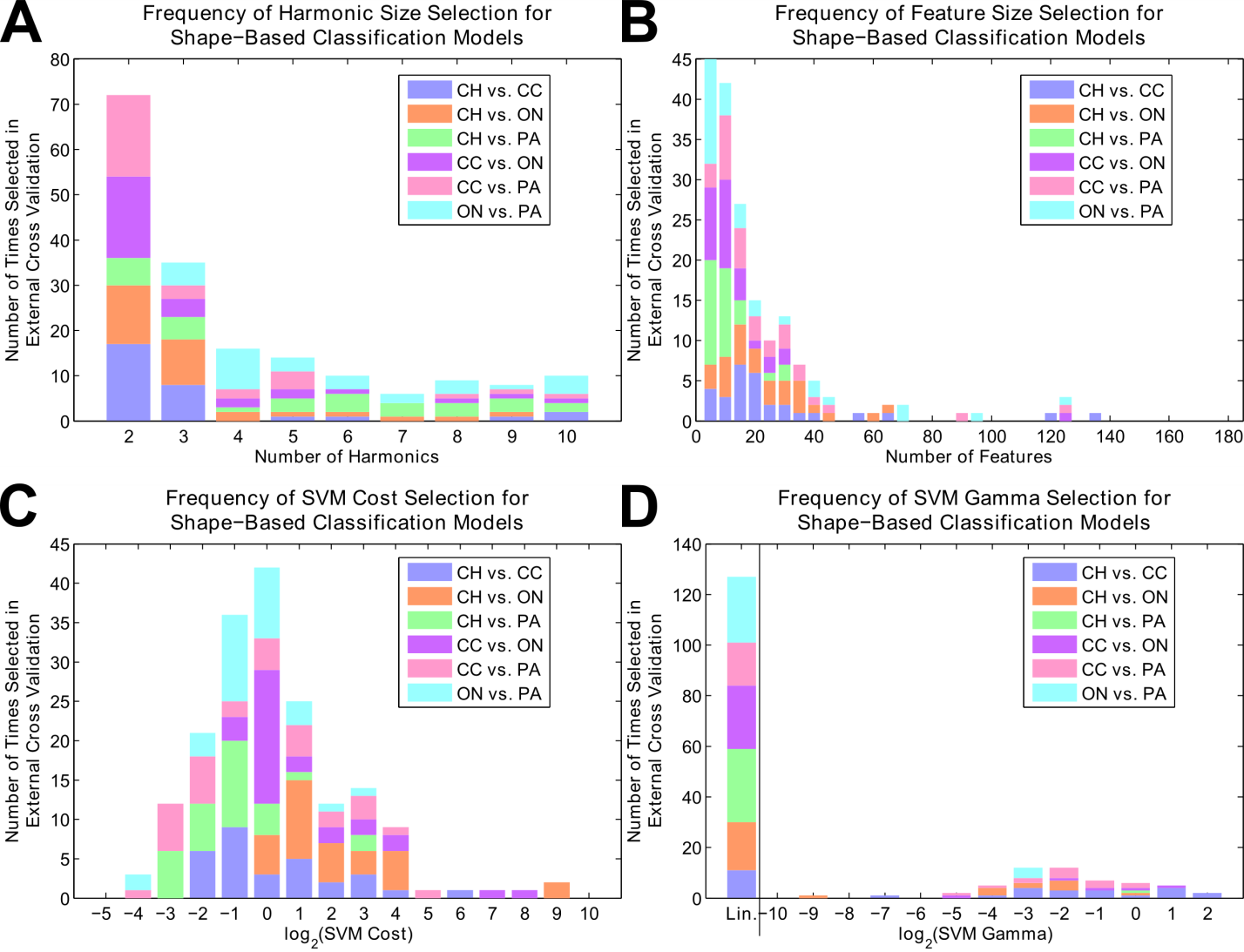


Distribution of a) maximum number of harmonics considered, b) number of features selected, c) SVM cost selected, and d) SVM gamma selected over 10 iterations times three folds of parameter estimation for various binary endpoints. The total length of each bar is a summation of counts over all binary endpoints while each color indicates count for a specific binary comparison. We can observe that, in most cases, the number of harmonics and features selected are less than four and twenty respectively. Moreover, SVM cost is less than 0 (in log2 scale) and linear classifiers are preferred over non-linear radial basis classifiers.
